# Patients With APECED Have Increased Early Mortality Due to Endocrine Causes, Malignancies and infections

**DOI:** 10.1210/clinem/dgaa140

**Published:** 2020-03-18

**Authors:** Joonatan Borchers, Eero Pukkala, Outi Mäkitie, Saila Laakso

**Affiliations:** 1 Children’s Hospital, Pediatric Research Center, University of Helsinki and Helsinki University Hospital, Helsinki, Finland; 2 Folkhälsan Research Center, Helsinki, Finland; 3 Research Program for Clinical and Molecular Metabolism, Faculty of Medicine, University of Helsinki, Helsinki, Finland; 4 Faculty of Social Sciences, Tampere University, Tampere, Finland; 5 Finnish Cancer Registry – Institute for Statistical and Epidemiological Cancer Research, Helsinki, Finland; 6 Department of Molecular Medicine and Surgery, Karolinska Institutet, and Clinical Genetics, Karolinska University Hospital, Stockholm, Sweden

**Keywords:** APECED, APS-1, mortality, cause of death

## Abstract

**Context:**

Autoimmune polyendocrinopathy-candidiasis-ectodermal dystrophy (APECED) is an autoimmune endocrinopathy with severe and unpredictable course. The impact of APECED on mortality has not been determined.

**Objective:**

To assess overall and cause-specific mortality of patients with APECED.

**Design and Setting:**

A follow-up study of Finnish patients with APECED from 1971 to 2018. Causes and dates of death were collected from Finnish registries.

**Patients:**

Ninety-one patients with APECED.

**Main Outcome Measure:**

Overall and cause-specific standardized mortality ratios (SMRs) determined by comparing the observed numbers of death and those expected on the basis of respective population death rates in Finland.

**Results:**

The overall disease mortality was significantly increased (29 deaths, SMR 11; 95% confidence interval [CI] 7.2-16; *P* < 0.001). The relative risk (SMR) was highest in the youngest age groups but the absolute excess risk was similar (about 10 per 10 000 person-years) in all age categories. The highest SMRs were seen for endocrine and metabolic diseases (SMR 570; 95% CI, 270-1000; *P* < 0.001) and for oral and esophageal malignancies (SMR 170; 95% CI, 68-360; *P* < 0.001). Mortality was also increased for infections, diseases of digestive system, alcohol-related deaths, and for accidents. Due to the small number of cases we were unable to evaluate whether mortality was affected by disease severity.

**Conclusions:**

Patients with APECED have significantly increased mortality in all age groups. Highest SMRs are found for causes that are directly related to APECED but also for infections. Increased alcohol- and accident-related deaths may be influenced by psychosocial factors.

Autoimmune polyendocrinopathy-candidiasis-ectodermal dystrophy (APECED, OMIM #240300), also known as autoimmune polyendocrine syndrome 1 (APS-1) is a rare, monogenic autosomal recessive disease. It is caused by mutations in the Autoimmune Regulator (*AIRE*) gene located in chromosome 21q22.3 (1). AIRE participates in the regulation of self-tolerance of the developing T-cells in the thymus (2). Defective function of AIRE results in production of multiple anti-cytokine and organ-specific antibodies, and leads to a severe autoimmune disease affecting multiple endocrine organs and other tissues (3, 4).

More than 20 different clinical manifestations have been associated with the disease, and the highly variable presentation makes the course of the disease unpredictable. The first clinical manifestations are often diagnosed in the childhood and new manifestations may appear throughout the lifetime (5-10). The disease was classically diagnosed when 2 of the 3 most common manifestations, chronic mucocutaneous candidiasis (CMC), hypoparathyroidism (HP), and primary adrenal insufficiency (PAI), were present or when one of the common manifestations were present in an individual with a sibling diagnosed with APECED (7). Today, APECED is often diagnosed by *AIRE* gene sequencing after initial manifestations, before the clinical criteria are fulfilled.

Only a few studies have described the mortality among patients with APECED by reporting the causes of death and age at death. In these studies, age at death varied from 3 to 64 years and median age at death from 5 to 34 years (10-14). According to the follow-up studies, the most common causes of death include hypocalcemia and adrenal crisis, oral and esophageal squamous cell carcinoma, and acute hepatitis (12-15). However, causes of death and mortality rates in comparison to the general population have not been systematically studied.

The Finnish APECED cohort is one of the largest worldwide, and the patients have been carefully followed, mostly in tertiary centers, for decades. This unique setting gave us a possibility to carry out a systematic study on cause-specific mortality. We hypothesized that APECED-related causes would especially carry increased mortality rates compared with the general population. We also studied whether disease characteristics at an early stage of APECED would affect the mortality.

## Methods

### Patients and research permits

The Finnish cohort of patients with APECED formed the basis of the study (16, 17). The cohort was initially recruited from university and central hospitals by contacting the respective endocrine and pediatric endocrine units. The cohort was later complemented by continuous recruitment of subsequently diagnosed patients from tertiary pediatric and adult endocrine centers throughout the country. The study was approved by the Research Ethics Committee of the Hospital District of Helsinki and Uusimaa. Informed written consent was obtained from all study participants or their guardians (for subjects aged < 18 years) at inclusion in the study.

### Data collection for clinical manifestations and causes of death

Beginning of the follow-up was defined for each subject as the date when the patient for the first time was introduced to the Finnish APECED cohort, or the beginning of 1971, whichever was later. End of the follow-up was the date of death, emigration, or the end of 2018, whichever was first. We collected the patient records from the hospitals in charge of the patients’ treatment and follow-up. From these patient records we extracted the age at diagnosis for the following disease manifestations: CMC, HP, PAI, diabetes, hypogonadism, hypothyroidism, growth hormone deficiency, hepatitis, intestinal dysfunction, exocrine pancreatic insufficiency, nephritis, alopecia, vitiligo, keratopathy, enamel dysplasia, and rash with fever.

Out of all 93 patients in the APECED cohort, 2 subjects who were deceased before 1971 were excluded. Dates and causes of death were collected from the registry maintained by Statistics Finland or from the patients’ death certificates, using the Finnish personal identity codes as patient identifiers. Classification of cause of death is based on medical or forensic evidence, which provides grounds for death certification. Forensic determination is necessary if the patient died at home from an unknown cause, if death is not due to illness, if it is accidental or violent, or caused by a treatment procedure or occupational disease. Statistics Finland has combined a 54-group short list that categorizes causes of death (18). Causes of death were also collected as International Classification of Disease (ICD) codes to further specify the causes of death.

### Standardized mortality ratio (SMR) calculations

For the statistical analysis, we counted overall and cause-specific numbers of deaths observed in the cohort and person-years at risk, by 5-year age groups, separately for males and females, and for eight 6-year calendar periods between 1971 and 2018. We calculated the expected numbers of deaths by multiplying person-years in each stratum by the corresponding mortality rate in the population of Finland. Absolute excess risk (AER) in each age group was defined as ratio of excess number of deaths and person-years. To calculate SMRs for broader age ranges, we added up age-specific observed numbers of deaths and divided by the sum of expected numbers of respective age categories. The 54-group short list of causes of death by Statistics Finland was used in the SMR calculations.

For additional stratifications, we used the number of clinical manifestations in the beginning of the follow-up (≤ 3 or > 3 components). We also divided the patients into groups depending on whether they had HP and/or PAI in the beginning of follow-up. To calculate 95% confidence intervals (CIs) for the SMRs, we assumed that the number of observed deaths followed a Poisson distribution.

## Results

### Patient characteristics

Altogether 46 female and 45 male Finnish patients with APECED were included in the present nationwide study (Table 1). This study population is likely to comprise the vast majority of the Finnish patients with APECED. The patients’ mean age at the beginning of follow-up was 12.3 years (median 10.5 years; range, 0.7-42.7) and mean follow-up time 27.1 years (median 27.0 years; range, 0.6-48.5). All patients were younger than 75 years at the end of the follow-up period.

**Table 1.** Number of Patients (*n*) in the Study, by Age, Number of APECED Manifestations and Hypoparathyroidism (HP) and/or Primary Adrenal Insufficiency (PAI) Status at the Beginning of Follow-Up. Person-Years Given by Dynamic Age at Follow-Up, 1971-2018

	Female		Male		Total	
Category	*n*	Person-years	*n*	Person-years	*n*	Person-years
Total	46	1301	45	1159	91	2458
Age, years						
0-14	37	206	35	197	72	403
15-29	9	493	6	468	15	961
30-44	-	390	4	331	4	720
45-59	-	198	-	141	-	339
60-74	-	14	-	22	-	35
≤ 3 manifestations	32	939	27	670	59	1609
> 3 manifestations	14	361	18	459	32	820
HP+ PAI-	23	656	13	372	36	1028
HP- PAI+	-	-	16	426	16	426
HP+ PAI+	21	597	10	208	31	805
HP- PAI-	2	46	6	152	8	198

Thirty-two of the 91 patients (35%) had more than 3 clinical manifestations in the beginning of the follow-up period. Altogether, 67 (74%) patients had HP. PAI was present in 47 (52%), and 31 (34%) also had HP at the beginning of the follow-up period (Table 1). Eight (9%) patients did not have either HP or PAI in the beginning of the follow-up.

Of the included 91 patients, 29 (32% females) had died during 1971-2018. Altogether, 25 deaths were due to diseases and 4 due to accidents. The median age at death for all patients was 35.0 years (range, 11.0-62.8 years). The median age at death was 36.7 years (range, 11.0-62.8 years) for patients deceased of disease-related causes and 18.1 years (range, 13.4-43.9) for patients deceased of accidents. Altogether 21 patients (72%) had died before 45 years of age. The median age at death for females was 34.6 years (range, 13.0-56.9 years) and for males 35.9 years (range, 11.0-62.8 years).

### Overall mortality

**Figure 1.**
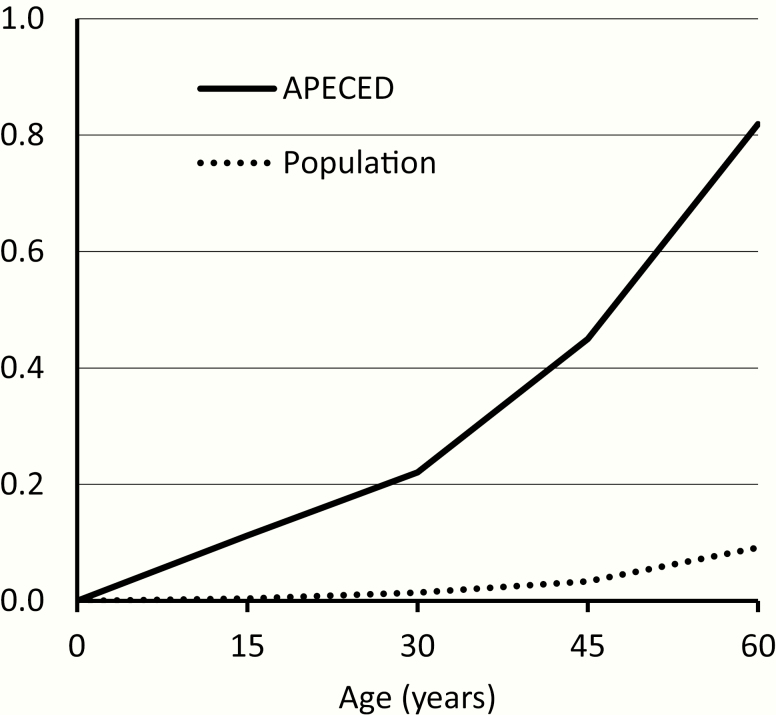
Cumulative all-cause mortality in the cohort of patients with APECED and in the Finnish general population with similar sex and follow-up period distribution. The cumulative values are calculated based on mortality rates for 15-year age groups.

The SMRs for diseases and accidents were both about 10-fold increased (Table 2). The SMRs for overall mortality were significantly elevated and the absolute excess risks were 7- to 14-fold in all age groups (Table 2). The cumulative mortality rate up to age 60 exceeded 80% in patients with APECED while the respective rate in the general population was less than 10% (Fig. 1).

**Table 2.** Observed (Obs) and Expected (Exp) Number of Deaths and Standardized Mortality Ratios (SMRs) With 95% Confidence Intervals (CIs) for All Causes, All Diseases and Accidents in the Whole Cohort and in Different Age Groups of Patients With APECED. For All-Cause Mortality, Also Shown Absolute Excess risks (AER) Per 1000 Person-Years

	All diseases				Accidents				All causes				
Age at follow-up	Obs	Exp	SMR	95% CI	Obs	Exp	SMR	95% CI	Obs	Exp	SMR	95% CI	AER
0-14	2	0.06	33	4.0-120 **	1	0.04	25	0.63-140	3	0.10	30	6.2-87 ***	7.2
15-29	5	0.19	26	8.4-61 ***	2	0.47	4.2	0.51-15	7	0.67	10	4.2-22 ***	6.6
30-44	10	0.54	18	8.9-34 ***	1	0.39	2.6	0.06-14	11	0.94	12	5.9-21 ***	14
45-59	5	1.07	4.7	1.5-11 **	-	0.23	0.0	0.0-16	5	1.30	3.8	1.3-9.0 *	11
60-74	3	0.39	7.7	1.6-22 *	-	0.03	0.0	0.0-120	3	0.43	7.1	1.5-21 *	7.3
Total	25	2.25	11	7.2-16 ***	4	0.56	7.2	2.0-18 *	29	3.43	8.5	5.7-12 ***	10

* *P* < 0.05; ** *P* < 0.01; *** *P* < 0.001.

The SMR for all causes including accidents was 8.5 (95% CI, 5.7-12; *P* < 0.001). The SMR for all diseases was 11 (95% CI, 7.2-16; *P* < 0.001) and for accidents, 7.2 (95% CI, 2.0-18; *P* < 0.05). No deaths due to suicide were reported. The accidents were classified as accidental fall, water transport accident, and accidental poisoning (non-alcohol).

The highest relative increase in the mortality was found in the youngest age groups < 45 years of age (Table 2). Significantly increased SMRs were found in all age groups for overall mortality and for disease-related mortality.

### Disease-specific mortality

The cause-specific SMRs, by gender, are reported in Table 3. SMRs for endocrine and metabolic diseases other than diabetes (570; 95% CI, 270-1000) and for oral and esophageal malignancies (170; 95% CI, 68-360) were significantly increased (Table 3). No deaths due to diabetes were reported. SMRs for infections (36; 95% CI, 6.4-110, one death in each gender) and for diseases of digestive system (37; 95% CI, 4.5-130; 2 deaths in males) were increased. According to ICD-classification, 1 death in the category of diseases of digestive system was due to hepatitis and the other due to inguinal hernia, which led to aspiration after surgery. Due to imprecise estimates, we were unable to evaluate whether mortality due to circulatory system and neurological diseases was different in the APECED cohort than in the general population. The SMR for alcohol-related deaths was significantly increased (8.0; 95% CI, 1.7-23).

**Table 3.** Observed (Obs) and Expected (Exp) Number of Deaths and Standardized Mortality Ratios (SMRs) With 95% Confidence Intervals (CIs) in Different Categories of Causes of Death Among Finnish Patients With APECED

	Female				Male				Total			
Cause of death	Obs	Exp	SMR	95% CI	Obs	Exp	SMR	95% CI	Obs	Exp	SMR	95% CI
**All diseases**	9	0.78	12	5.3-22 ***	16	1.48	11	6.2-18 ***	25	2.25	11	7.2-16 ***
Endocrine and metabolic diseases^1^	4	0.01	480	130-1200 ***	6	0.01	650	240-1400 ***	10	0.02	570	270-1000 ***
Malignant neoplasms	1	0.35	2.9	0.07-16	4	0.38	11	2.9-27 **	5	0.73	6.9	2.2-16 **
Oral and esophageal malignancy	1	0.01	140	7.1-650 *	4	0.02	180	63-420 ***	5	0.03	170	69-360***
Diseases of digestive system	-	0.02	0.0	0.0-190	2	0.03	58	7.0-210 **	2	0.05	37	4.5-130 **
Infectious diseases^2^	1	0.02	52	2.7-250 *	1	0.04	27	1.4-130 *	2	0.06	36	6.4-110 **
Diseases of the circulatory system	-	0.13	0.0	0.0-28	2	0.52	3.8	0.46-14	2	0.65	3.1	0.37-11
Neurological diseases	1	0.04	24	0.61-130	-	0.05	0.0	0.0-69	1	0.09	11	0.27-59
All alcohol related deaths	2	0.10	20	2.5-73 *	1	0.28	3.6	0.09-20	3	0.38	8.0	1.7-23 *
**Accidents** ^3^	1	0.12	8.2	0.21-46	3	0.44	6.9	1.4-20 *	4	0.56	7.2	2.0-18 *

* *P* < 0.05; ** *P* < 0.01; *** *P* < 0.001; ^1^ Diabetes excluded; ^2^ HIV and tuberculosis excluded; ^3^ Alcohol related accidents excluded.

There were no significant differences in SMRs for all diseases, endocrine and metabolic diseases, and oral and esophageal malignancies between subgroups stratified by the number and spectrum of manifestations at the beginning of follow-up (Table 4).

**Table 4.** Observed (Obs) and Expected (Exp) Number of Deaths and Standardized Mortality Ratios (SMRs) with 95% Confidence Intervals (CIs) for All Diseases and to Most Common Causes of Death. Patients are Divided in Subgroups Depending on Characteristics of the Disease in the Beginning of Follow-Up

	All diseases				Endocrine and metabolic diseases				Oral and esophageal malignancy			
Subgroup	Obs	Exp	SMR	95% CI	Obs	Exp	SMR	95% CI	Obs	Exp	SMR	95% CI
≤3 manifestations	13	1.42	9.2	4.9-16 ***	4	0.01	340	93-870 ***	3	0.02	160	44-420 ***
>3 manifestations	12	0.83	14	7.4-25 ***	6	0.01	1000	380-2200 ***	2	0.01	190	34-590 ***
HP + PAI-	8	0.79	10	4.4-20 ***	4	0.01	540	150-1400 ***	1	0.01	100	5.3-490 *
HP- PAI+	5	0.76	6.6	2.1-15 **	-	0.00	0.0	0.0-1000	2	0.02	170	30-520 ***
HP + and PAI+	11	0.55	20	10-36 ***	5	0.00	1000	320-2300 ***	2	0.01	360	64-1100 ***
HP- and PAI-	1	0.15	6.8	0.17-38	1	0.00	640	16-3600 **	-	0.00	0.0	0.0-1700

Abbreviations: HP, hypoparathyroidism; PAI, primary adrenal insufficiency. * *P* < 0.05; ** *P* < 0.01; *** *P* < 0.001.

## Discussion

This prospective cohort study reports the first systematic analysis of mortality and causes of death in APECED. In this study of 91 patients with APECED, we show that both the overall mortality and mortality for several disease-related causes were significantly increased. We also found a significant increase in the mortality from infections, alcohol-related causes, and accidents. We found the highest relative increase in the mortality in children and young adults, and most of the deaths occurred before the age of 45 years.

The majority of the causes of patient deaths seem to be related to APECED. We found the highest mortality rates and the highest number of deaths in endocrine diseases in both female and male patients. This finding is congruent with the previous studies that have reported several deaths due to endocrine causes, such as hypocalcemic and adrenal crisis (7, 12, 14). Mortality due to oral and esophageal malignancies was also strongly increased. Previous studies have shown increased risk of oral malignancies in patients with APECED. Altered T-cell function and oral candidiasis are reported to be probable factors increasing the risk for squamous cell carcinomas (15, 19). We also found increased mortality due to diseases of digestive system, based on deaths in male patients with APECED. One patient in this category had died of hepatitis, which has also previously been reported as a cause of death in APECED (7, 12, 14). Our findings confirm observations in earlier case series, reporting high number of deaths due to disease-related causes.

Importantly, our data also indicate for the first time, increased mortality due to infections in patients with APECED. There are few previous studies describing patients with severe infections and reporting deaths due to infections such as bacterial septicemias of various species, pneumonia, measles, and influenza A (7, 12, 14, 20-22). It has been hypothesized that anticytokine autoantibodies, found in most of the patients, and asplenia may play a role in predisposing to severe infections (23, 24). However, increased mortality due to infections has not been reported before. Our observation has significant clinical implications and warrants further research.

Mortality from alcohol-related deaths and accidents was also increased in patients compared with the general population. The reason for increased risk of fatal accidents is not clear. The 54 categories of causes of death include suicides as a distinct category but no suicides were reported. As the patients have a high risk of alcohol-related deaths, increased mortality appears to be affected by social and mental factors in addition to the clinical disease. In addition to the clinical disease manifestations, psychosocial factors also strongly affect mortality since many patients suffer from depression and hopelessness (9).

Our study shows that mortality in patients with APECED was increased already at an early age and was similarly increased in all age groups. Due to small number of cases we were unable to evaluate whether mortality was affected by disease severity. In previous studies, PAI has been associated with increased mortality attributed to malignancies, cardiovascular diseases, and infections (26, 27). On the contrary, no clear increase in mortality has been found in patients with hypoparathyroidism (28).

The low number of patients limits the power of the analyses. The cohort consisted of 91 patients, out of whom 29 had died by the end of 2018. Still, the Finnish cohort is one of the largest cohorts of patients with APECED, with a long follow-up. Thanks to the unique personal identity codes given to all residents in Finland since 1967 and the complete population registration system, the record linkages are accurate and there are no losses to follow-up (29). Even though there are potential sources of errors in the process of registering causes of death, according to validation surveys, the Finnish causes of death have been found appropriate to serve as reference data in SMR statistics (30). Because the observed and expected deaths are based on the same registry, possible inaccuracies in coding are not likely to affect SMR estimates.

In conclusion, our study shows that overall mortality and mortality from APECED-related causes at all ages are significantly increased compared with the Finnish population. These findings are in accordance with previous studies that have reported deaths due to the clinical manifestations of the disease. In addition, our results suggest increased mortality due to previously unreported causes, such as infections. These results emphasize the importance of careful monitoring of patients, with a special focus on endocrine manifestations, development of oral neoplasms, infections, and psychosocial factors.

## Abbreviations

AER: absolute excess risk
APECED: autoimmune polyendocrinopathy-candidiasis-ectodermal dystrophy
CMC: chronic mucocutaneous candidiasis
HP: hypoparathyroidism
PAI: primary adrenal insufficiency
SMR: standardized mortality ratio

## Additional Information

***Disclosure Summary:*** The authors have nothing to disclose.

***Data Availability:*** Restrictions apply to the availability of data generated or analyzed during this study to preserve patient confidentiality. The corresponding author will on request detail the restrictions and any conditions under which access to some data may be provided.
